# Encapsulated Mesenteric Fat Necrosis

**DOI:** 10.5334/jbr-btr.1066

**Published:** 2016-03-31

**Authors:** Isabelle De Kock, Louke Delrue

**Affiliations:** 1 Department of Radiology, Ghent University Hospital, Ghent, Belgium

**Keywords:** abdominal pain, fat-containing lesion, fat necrosis, ultrasound, CT

A 33-year-old female presented at the emergency department with a 36-hour history of peri-umbilical pain associated with anorexia and nausea. On physical examination, she was tender to palpation in the right lower quadrant. Psoas sign was absent, and there was no rebound tenderness. Laboratory examination revealed slightly elevated neutrophil count and elevated C-reactive protein. Her past medical history was unremarkable.

Appendicitis was suspected and abdominal ultrasound was performed, which demonstrated an oval hyperechogenic mass with a hypo-echogenic rim in the right para-umbilical region (Figure A). There was maximal probe tenderness at that point. The appendix was not well seen. Subsequent CT showed a round, encapsulated mass with predominantly fat attenuation located in the right para-umbilical region (Figures B and C). There were no calcifications present within the mass. No enhancement was seen after intravenous contrast administration. The appendix appeared normal.

**Figure 1:**
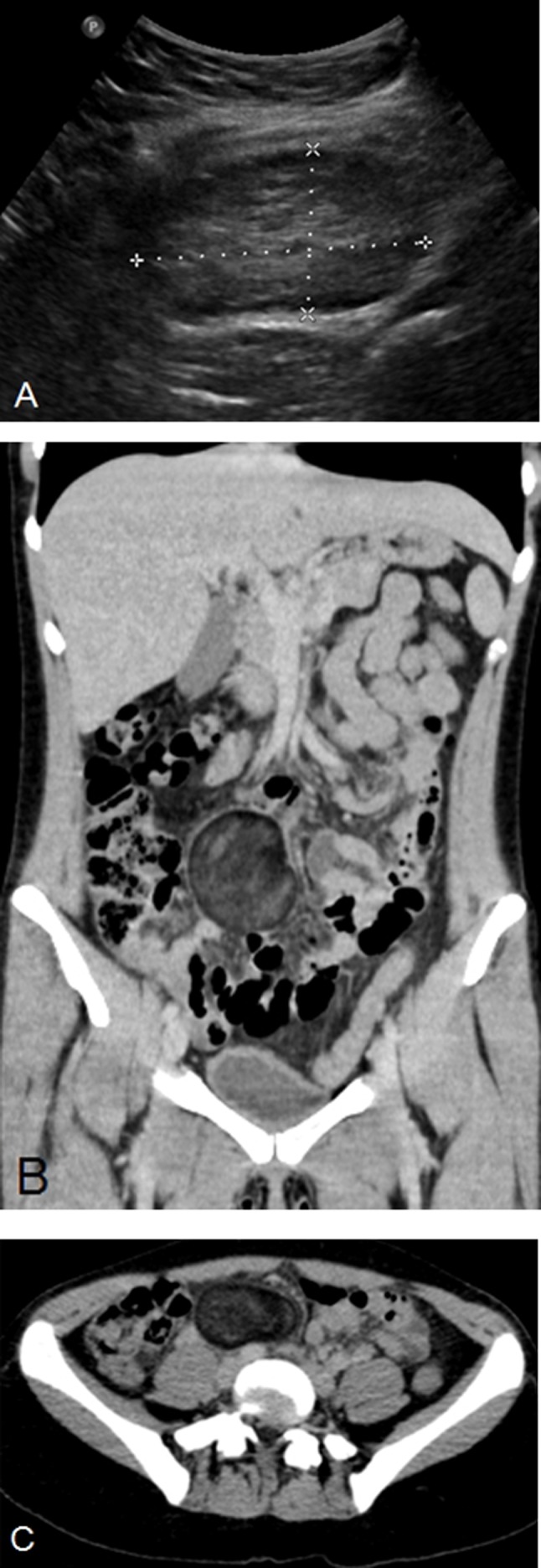


These imaging findings, associated with the clinical presentation of the patient, led to the diagnosis of encapsulated mesenteric fat necrosis.

## Comment

Encapsulated fat necrosis is an entity which falls within the umbrella term of ‘intraperitoneal focal fat infarction’ (IFFI). Other and more common types of IFFI include torsion and infarction of the greater omentum or epiploic appendages. Although first described in the breast in 1975, encapsulated fat necrosis may occur anywhere in the body. When located in the abdomen, fat necrosis may cause abdominal pain and have a clinical manifestation that mimics that of acute abdomen. The pathogenic mechanisms causing encapsulated fat necrosis are unknown. However, trauma and ischemia are speculated to be the main histogenetic factors causing infarction of adipose tissue lobules. Subsequently, a fibrous capsule is formed around the necrotic fatty tissue separating these lesions from the surrounding tissues. This capsule is the hallmark of encapsulated fat necrosis. The interior contents of the fibrous capsule contain necrotic adipose tissue with occasional inflammation and calcification.

The most important differential diagnosis is liposarcoma. Encapsulated fat necrosis can demonstrate a mild mass effect on adjacent structures, and its fibrous capsule may slightly enhance after administration of intravenous contrast material: findings that raise suspicion of a malignant lesion. However, unlike liposarcoma, fat necrosis does not show organ invasion and may be focally tender at palpation. Furthermore, liposarcoma is one of the most common primary neoplasms in the retroperitoneum; primary mesenteric and primary peritoneal liposarcomas are rare. Also, encapsulated fat necrosis is likely to decrease in size over time, whereas liposarcomas usually get larger over time [1].

Encapsulated fat necrosis should be included in the differential diagnosis of abdominal fat-containing lesions. Because fat necrosis and malignant processes such as liposarcoma may mimic one another, clinical history and prior imaging studies are essential for accurate diagnosis.

## Competing Interests

The authors declare that they have no competing interests.
